# Added sugar intake and its associations with incidence of seven different cardiovascular diseases in 69,705 Swedish men and women

**DOI:** 10.3389/fpubh.2024.1452085

**Published:** 2024-12-09

**Authors:** Suzanne Janzi, Esther González-Padilla, Stina Ramne, Sara Bergwall, Yan Borné, Emily Sonestedt

**Affiliations:** ^1^Nutritional Epidemiology, Department of Clinical Sciences Malmö, Lund University, Malmö, Sweden; ^2^Novo Nordisk Foundation Center for Basic Metabolic Research, Faculty of Health and Medical Sciences, University of Copenhagen, Copenhagen, Denmark; ^3^Vascular Diseases, Department of Clinical Sciences Malmö, Lund University, Malmö, Sweden

**Keywords:** added sugar, cardiovascular disease, dietary sugars, sugar-sweetened beverages, heart disease

## Abstract

**Introduction:**

The adverse health effects of sugar-sweetened beverage intake are well-established, but the implications of overall added sugar intake remain unclear. We investigated the associations between intake of added sugar and various sugar-sweetened foods and beverages and risk of 7 cardiovascular diseases in 69,705 participants aged 45–83 years (47.2% female) from the Swedish Mammography cohort and Cohort of Swedish men.

**Methods:**

Questionnaire-based diet and lifestyle assessments were administered in 1997 and 2009. National registers were used for ascertainment of ischemic stroke (*n* = 6,912), hemorrhagic stroke (*n* = 1,664), myocardial infarction (*n* = 6,635), heart failure (*n* = 10,090), aortic stenosis (*n* = 1,872), atrial fibrillation (*n* = 13,167), and abdominal aortic aneurysm (*n* = 1,575) until December 31st, 2019. The associations were studied using Cox regression with time-updated exposure and covariate data.

**Results:**

Added sugar intake was positively associated with ischemic stroke and abdominal aortic aneurysm, although the highest risks of most outcomes were found in the lowest intake category. Positive linear associations were found between toppings intake and abdominal aortic aneurysm, and between sweetened beverage intake and ischemic stroke, heart failure, atrial fibrillation, and abdominal aortic aneurysm. Negative linear associations were found between treats intake (pastries, ice cream, chocolate, and sweets) and all outcomes, and between toppings intake (table sugar, honey, jams, and marmalades) and heart failure and aortic stenosis.

**Discussion:**

The findings suggest that the associations between added sugar intake and cardiovascular diseases vary by disease and source of added sugar. The findings emphasize the adverse health effects of sweetened beverage consumption and indicate higher cardiovascular diseases risks with lower treats intake, which warrants further investigation.

## Introduction

Cardiovascular disease (CVD) comprises various diseases of the heart and blood vessels and is currently the leading cause of death and disease burden in Europe, primarily by stroke and ischemic heart disease (1, 2). Despite diet being one of the main modifiable risk factors of many CVDs (2), the evidence regarding added sugar intake and CVD risk is scarce and inconclusive (3–7). Furthermore, most studies have primarily focused on sugar-sweetened beverage consumption rather than overall added sugar intake even though sugar-sweetened beverages make up only ~14% of added sugar intake in Sweden and ~25% in the United States (8, 9).

The guidelines for sugar intake vary between different authoritative bodies globally, both with regards to the recommendation thresholds and the basis of the guidelines, with some being based mainly on micronutrient dilution (i.e., the displacement of nutrient-dense food intake by overconsumption of energy-dense foods poor in nutrients), while others are based mainly on the established risks of caries and overweight (6). The Nordic nutrition recommendations recommend limiting added sugar and free sugar (all added sugar as well as naturally occurring sugars in honey, syrups, fruit juices, and fruit juice concentrates) intake to < 10% of energy intake (E%), based on the risk of micronutrient dilution and developing chronic metabolic diseases and dental caries (10). The American dietary guidelines have a similar recommendation for added sugar intake based on the risk of micronutrient dilution, overweight and obesity, as well as related chronic metabolic diseases (11), and The World Health Organization has a similar recommendation for free sugar intake based on the risk of dental caries and overweight. The World Health Organization also further suggest reducing the intake to below 5 E% for additional benefits for dental health (12).

The European Food Safety Authority's (EFSA) scientific opinion on a tolerable upper intake level for dietary sugars published in 2022 (6) reported moderate evidence for a dose-response relationship between added and free sugar intake and obesity and dyslipidemia, as well as evidence of high certainty pointing toward associations between intake of sugar-sweetened beverages and obesity, type 2 diabetes, hypertension, and CVD incidence. However, no conclusion could be drawn about the associations between added and free sugar intake and CVD risk due to the lack of studies available (6).

Most studies on added sugar intake and CVD risk have studied CVD as a composite endpoint, but previous findings of ours indicate that the association between added sugar and CVD incidence varies between different CVDs (13), thus highlighting the importance of studying different CVDs separately. Research on the associations between added sugar intake and most individual CVDs such as for example abdominal aortic aneurysm and atrial fibrillation is lacking, and the very few studies that have investigated the associations with for example myocardial infarction or stroke present inconsistent findings (13–15). In addition to the lack of studies investigating the long-term effects of added sugar intake and the insufficient evidence regarding overall added sugar intake and CVD risk (3, 16), many studies only include a single dietary assessment at baseline, meaning that the assumption has to be made that this single assessment is representative of the participants' diets over time. In this study, diet was assessed at two time-points (in years 1997 and 2009) for the majority of the participants.

The purpose of this study was therefore to examine whether there are associations between intake of added sugar, sugar-sweetened foods and beverages, and risk of several types of CVD (i.e., ischemic stroke, hemorrhagic stroke, myocardial infarction, heart failure, aortic stenosis, atrial fibrillation, and abdominal aortic aneurysm) among 69,705 participants in two Swedish cohorts, with time-updated data for a majority of the participants in the study. Discerning whether there are associations between added sugar intake and CVD risk could help inform future nutritional recommendations.

## Methods

### Study population

The study population consists of female participants from the Swedish Mammography Cohort (SMC) and male participants from the Cohort of Swedish Men (COSM), both of which are population–based prospective cohort studies in Central Sweden and part of the national research infrastructure SIMPLER (Swedish Infrastructure for Medical Population-based Life-course Environmental Research). Invitations were sent by mail to all women born between 1914–1948 living in Uppsala county and Västmanland county, and to all men born between 1918–1952 living in Örebro county and Västmanland county (17).

The participants of SMC and COSM completed identical questionnaires (except for some sex-specific questions) regarding diet, health, and lifestyle factors in the years 1987–1990 (SMC only), 1997, 2008, and 2009. Ultimately, 66,651 women participated in SMC in 1987–1990 (74% response rate). During 1997, which serves as the baseline of our study as it was the first assessment that included both cohorts, the response rate was 70% of the still living participants from 1987 in SMC (*n* = 39,227), and 49% in COSM (*n* = 48,850) (17). The study was approved by The Swedish Ethical Review Authority (dnr: 2019-03986) and completion of the self-administered questionnaire was considered to convey informed consent.

**Figure 1:**
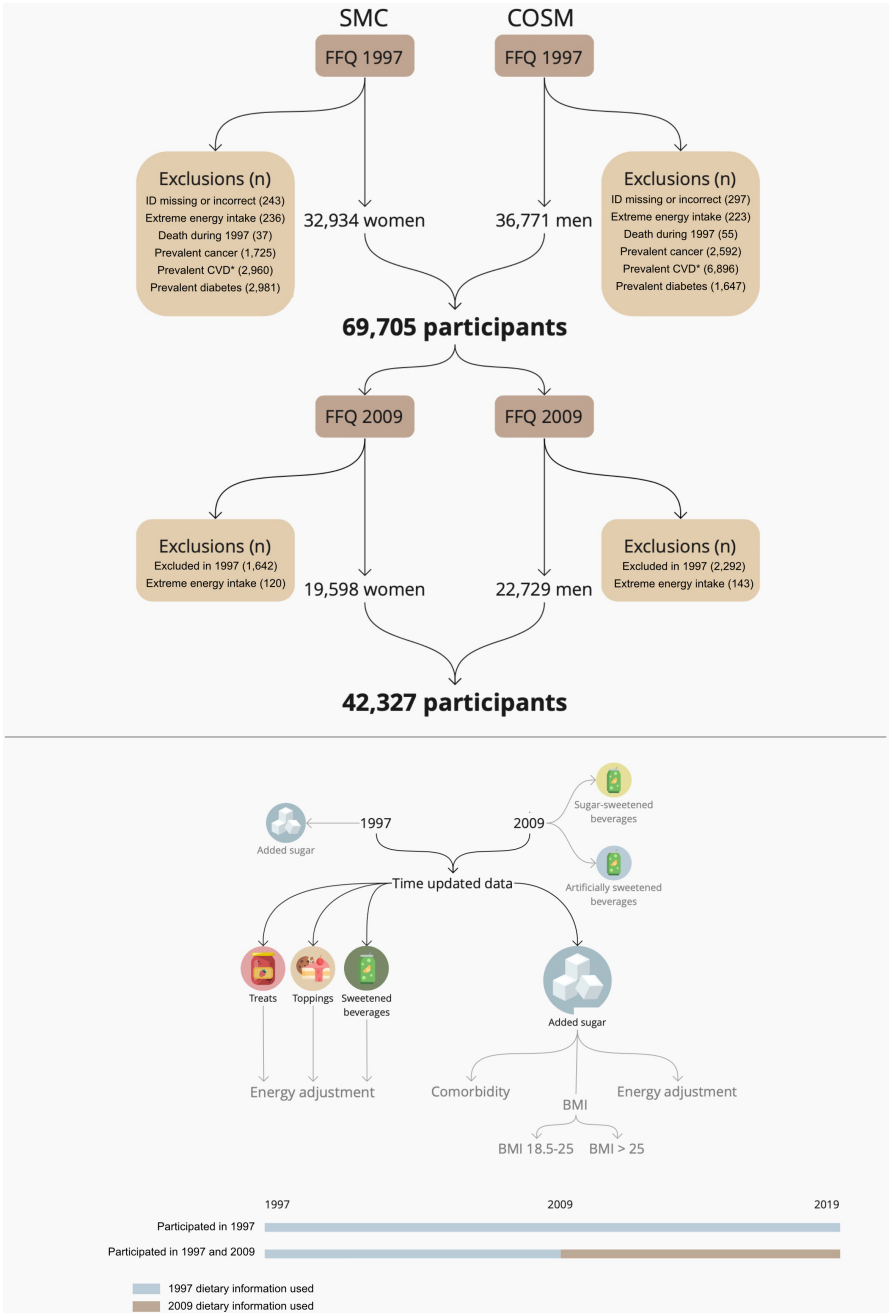
Description of the study population, exclusions, and statistical analyses (with the black arrows representing the main analyses, and the gray arrows representing the sensitivity analyses). *Prevalent cases of ischemic stroke, intracerebral hemorrhage, subarachnoid hemorrhage, myocardial infarction, heart failure, atrial fibrillation, aortic valve stenosis, and abdominal aortic aneurysm. BMI, Body Mass Index; COSM, Cohort of Swedish men; SMC, Swedish mammography cohort; FFQ, Food frequency questionnaire; CVD, Cardiovascular disease.

In 2008, all participants of SMC and COSM were sent a health and lifestyle questionnaire, and those who responded received an expanded semiquantitative food frequency questionnaire (FFQ) in 2009. In total, 47,918 participants responded to the 2009 questionnaire, and 42,327 participants (19,598 women and 22,729 men) remained after baseline exclusions (61% of participants from 1997) (Figure 1).

### Baseline exclusions

As SMC originally only included women without cancer, participants in both cohorts with cancer recorded in Swedish registers prior to the baseline were excluded from the present study. Further exclusions were made of those with prevalent CVD (identified using national registers), self-reported diabetes at baseline, death prior to January 1^st^, 1998, missing or incorrect ID, and those deemed to have had extreme energy intakes, since it could indicate misreporting. Extreme energy intake was defined as being outside of 3 standard deviations (SDs) below and above the log_e_-transformed mean energy intake, corresponding to below 470 kcal and above 12,790 kcal in COSM and below 263 kcal and above 9,647 kcal in SMC. Thus, 32,934 participants from SMC and 36,771 participants from COSM remained (69,705 participants in total) in 1997. For the assessment in 2009, individuals deemed to have had extreme energy intakes (i.e., outside of 3 SDs below and above the log_e_-transformed mean energy intake, corresponding to below 580 kcal and above 5,687 kcal in SMC and below 787 kcal and above 7,932 kcal in COSM) were excluded, resulting in 19,598 participants in SMC and 22,729 participants in COSM remaining. In total, 42,327 participants responded to the 2009 questionnaire and remained after exclusions (Figure 1).

### Dietary assessment

Dietary information at baseline (in 1997) was obtained using a 96-item validated semi-quantitative FFQ covering average intakes of various foods and beverages during the preceding year. For all food items and for alcohol intake, the participants could choose between 8 or 9 predetermined consumption frequencies, and portion sizes were either specified as standardized portions or self-reported (as was the case for alcohol consumption). The questions regarding frequency of beverage consumption (including sweetened beverages, tea, and coffee) and table sugar and honey intakes were posed as open-ended questions about the number of daily or weekly servings consumed in the preceding year.

In 2009, a 132-item semi-quantitative FFQ was distributed to COSM and SMC participants in order to obtain updated dietary information. The 132-item FFQ included additional foods and was designed to capture the consumption of specific foods and diets that were consumed more frequently in 2009 than in 1997. Nutrient intakes were estimated by multiplying the consumption frequency of each food item with the nutrient content of sex- and age-specific portion sizes obtained from weighed food records in a subgroup of 152 men from COSM and 129 women from SMC. The nutrient values were derived from the Swedish Food Composition Database (18). Participants with missing information about individual foods were interpreted as non-consumers.

The 1997 dietary assessment was validated against 14 24-h recall interviews among 248 Swedish men living in central Sweden, randomly selected from the Swedish population register, with a Spearman rank correlation coefficient of 0.70 being reported for sucrose (19). Further, a validation study was carried out among 129 randomly selected women from SMC using four 1-week records that were completed 3–4 months apart. The reported correlation coefficients were 0.6 for sweetened beverages, 0.5 for jams and marmalades, and 0.4 for sweets (A. Wolk, unpublished observations, 1992).

#### Added sugar estimation

The participants' total added sugar intakes were estimated by subtracting their intakes of naturally occurring sugars in fruit, vegetables, fruit juices, and jams from their total intake of sucrose and monosaccharides. The amounts of naturally occurring monosaccharides and sucrose were estimated using the Swedish Food Composition Database (18) (Supplementary Table S1). The added sugar intakes (g/day) were then converted into percentages of non-alcoholic energy intakes. This added sugar density variable was further categorized into ≤ 5 E%, >5–7.5 E%, >7.5–10 E%, >10–15 E%, >15–20 E%, and >20 E%. The categories were designed to allow a wide range of sugar intakes to be studied, including those commonly used in nutritional recommendations as well as extreme intakes.

#### Sugar-sweetened foods and beverages

As the macronutrient distribution, energy density, and consumption patterns vary between different sources of added sugar, sugar-sweetened foods and beverages were studied in groups of treats (pastries, ice cream, sweets, and chocolate), toppings (table sugar, honey, jams, and marmalades), and sweetened beverages (all sweetened sodas and fruit drinks but not pure fruit juices). Notable differences are that toppings generally have negligible fat and protein contents, whereas treats tend to have a higher fat content, and we additionally hypothesized that treats were more likely to be overconsumed than toppings. As liquid sugar has been suggested to be metabolized differently than solid sugar and to consequently cause different health outcomes (20), a category of sweetened beverages was also created. The dietary questionnaire from 1997 asked about overall sweetened beverage intake, while the 2009 questionnaire differentiated between sugar-sweetened beverages and artificially sweetened beverages, which were combined to create the sweetened beverage variable in the 2009 data. Participants with missing information about individual foods were interpreted as non-consumers (Supplementary Table S2).

Intake of sweetened beverages was divided into categories of ≤ 1, >1–3, >3–5, >5–8, and >8 servings/week, intake of treats was divided into categories of ≤ 2, >2–5, >5–8, >8–14, and >14 servings/week, and intake of toppings was divided into categories of ≤ 2, >2–7, >7–14, >14–28, and >28 servings/week. The categories for the sugar-sweetened foods and beverages were set based on categories previously used in two other Swedish cohorts carried out around the same period as the baseline of COSM and SMC, and were determined by examining the restricted cubic spline curves of association between added sugar intake and total mortality (21, 22).

### Case ascertainment

The participants were followed until diagnosis of the studied outcome, death, or the end of the follow-up period (December 31^st^, 2019), whichever occurred first. The participants' Swedish personal identification numbers were used to identify cases of the studied outcomes between 1998 and 2019 in the Swedish National Inpatient Register and the Cause of Death Register, according to the International Classification of Diseases 10 (ICD−10) and corresponding codes in earlier editions. The Swedish National Inpatient Register has been reported to have high diagnostic validity, with positive predictive values of >90% being reported for most of the studied outcomes (23). The studied outcomes and their ICD-10 codes were ischemic stroke (I63), myocardial infarction (I21), heart failure (I50 and I11.0), atrial fibrillation (I48), aortic valve stenosis (I35.0 and I35.2), and abdominal aortic aneurysm (I71.3 and I71.4). As it has been indicated that ischemic stroke incidence is affected more by lifestyle factors than hemorrhagic stroke (24), ischemic stroke cases and hemorrhagic stroke cases were studied separately, with the variable for hemorrhagic stroke including cases of intracerebral hemorrhage (I61) and subarachnoid hemorrhage (I60).

### Statistical analyses

All analyses were conducted using R version 4.0.3 (R Foundation for Statistical Computing, Vienna, Austria), with *P*–values < 0.05 denoting statistical significance. The population baseline characteristics were tested across the added sugar intake categories, with normally distributed continuous variables presented as mean with SD, skewed continuous variables presented as median with interquartile range (IQR), and categorical variables presented as number of participants with proportion of total participants.

Cox proportional hazards regression models were used to investigate the associations between the studied exposures and outcomes using the *survival* R package (25) with the time of follow-up being used as the time variable. Time-updated exposures and covariates were incorporated in the Cox regression for participants who responded to both the 1997 and 2009 questionnaires (*n* = 42,327). This was done by dividing the follow-up period into two intervals (1997–2009 and 2009–2019), with the 1997 data being used in the first interval and the updated data from 2009 being used in the second interval. For those who only completed the 1997 questionnaires (*n* = 27,378), the data from 1997 was used in both intervals (Figure 1). The *P*-values for the trends were analyzed using the medians of the exposure categories in the models, using the lowest intake category as a reference.

Associations between added sugar intake and incident disease outcomes were investigated using several different models that were adjusted for factors that are established risk factors for CVDs and are associated with added sugar intake. The first model was adjusted for age (years), sex, and total energy intake (kilocalories/day). The second model was additionally adjusted for lifestyle factors as categorical variables: smoking status [current, former, never, missing (n_1997_ = 1,086, n_2009_ = 1,218)], educational level [less than high school, high school, university, missing (n_1997_ = 279, n_2009_ = 88)], alcohol consumption (non-consumers and quintiles of sex-specific intakes), as well as walking/cycling [almost never, < 20 min/day, 20–40 min/day, 40–60 min/day, 1–1.5 h/day, >1.5 h/day, missing (n_1997_ = 6,084, n_2009_ = 676)], and exercise (type of exercise not specified) [ < 1 h/week, 1–2 h/week, 3–4 h/week or ≥5 h/week, missing (n_1997_ = 7,385, n_2009_ = 917)]. The smoking variable was modified so that all participants who indicated years since they stopped smoking were recorded as former smokers. The main model was additionally adjusted for body mass index (BMI) (kg/m^2^) and dietary factors, including consumption of processed meat (g/day), coffee (g/day), as well as energy adjusted saturated fatty acid and fiber intakes (using the nutrient residual model). The proportional hazards assumptions were tested using Schoenfeld residuals, and the models were stratified for age, sex, and walking/cycling to attain proportionality. Missing values were included as a separate category for each of the categorical variables, while missing values for continuous dietary covariates were interpreted as zero-consumption. Non-linear trends of CVDs were assessed using restricted cubic splines and the rms R package with 5 knots placed at the 5^th^, 27.5^th^, 50^th^, 72.5^th^, and 95^th^ percentiles of added sugar intake with 10 E% as reference (26).

#### Sensitivity analyses

Interactions between sex and added sugar intake, as well as BMI and added sugar intake, were studied by adding them as continuous interaction terms in the main model for each of the outcomes. For the associations with statistically significant interactions, stratified analyses were conducted.

Since the 1997 questionnaire only asked about overall sweetened beverage intake (i.e., sugar-sweetened beverages and artificially sweetened beverages combined), dietary data from 2009 was used to study consumption of artificially sweetened beverages and sugar-sweetened beverages separately, using 2009 as a baseline, among participants who responded to the 2009 questionnaire (*n* = 42,327).

Comorbidities are prevalent with many CVDs (27, 28). To take comorbidity into consideration, a sensitivity analysis was conducted in which all participants with incidence of any of the other studied CVDs prior to incidence of the outcome of interest were excluded from the analysis.

As there are several different methods for energy adjustment in epidemiological studies (29), sensitivity analyses were conducted using additional energy-adjustment methods. For added sugar, the residual method were used in addition to the nutrient density method (as used in the main analyses) (29). For sugar-sweetened foods and beverages, the nutrient density method and the residual method were used in addition to the standard multivariate method (as used in the main analyses). The energy-adjusted intakes using the nutrient density method and the residual method were standardized to the study population's mean energy intake.

To evaluate how the addition of the 2009 diet assessment affected the risk estimates in the main analyses for added sugar intake, a sensitivity analysis using only the 1997 baseline dietary data was conducted.

Further, to reduce bias by reverse causality, a sensitivity analysis was conducted where we excluded cases of the studied outcome that occurred within the first 3 years of the follow-up period.

Finally, to investigate how including missing data may have affected the results for added sugar, we conducted a sensitivity analysis in which only participants with complete data for all sugar-sweetened foods and beverages and categorical covariates were included. Similarly, we conducted sensitivity analyses for treats, toppings, and sweetened beverage intake, including only participants with complete data for the respective variables and categorical covariates.

## Results

### Study population characteristics

The study population consisted of 69,705 participants (47.2% female), with a mean BMI of 25.3 kg/m^2^, mean age of 59.9 years (ranging between 45 and 83 years) and mean added sugar intake of 9.1 E% at baseline (Table 1). Higher added sugar consumers tended to be male, with higher exercise levels and with lower education levels than lower added sugar consumers. Furthermore, those consuming high amounts of added sugar were generally older, had higher energy intakes, and had higher intakes of toppings and sweetened beverages. The intake of treats was more evenly distributed across the added sugar intake groups than the other sugar-sweetened foods and beverages (Table 2).

**Table 1:** Reported dietary intakes in SMC and COSM in 1997 and 2009 shown in men and women separately as well as combined.

	**1997**			**2009**		
	**Men**	**Women**	**Overall**	**Men**	**Women**	**Overall**
	**(*****n** =* **36,771)**	**(*****n** =* **32,934)**	**(*****n** =* **69,705)**	**(*****n** =* **22,729)**	**(*****n** =* **19,598)**	**(*****n** =* **42,327)**
**Mean (SD)**						
Energy intake, *kcal/day*	2,702 (877)	1,746 (564)	2,251 (885)	2,649 (818)	1,919 (580)	2,311 (805)
Added sugar, *E%*	9.8 (5.5)	8.3 (5.0)	9.1 (5.3)	7.5 (4.3)	6.1 (3.7)	6.8 (4.2)
Carbohydrates, *E%*	50.0 (6.08)	49.6 (6.3)	49.8 (6.2)	46.1 (6.0)	44.9 (6.3)	45.5 (6.2)
Fat*, E%*	31.1 (5.3)	31.6 (5.5)	31.3 (5.4)	30.9 (5.1)	32.1 (5.3)	31.5 (5.2)
Protein, *E%*	15.7 (2.3)	16.6 (2.8)	16.1 (2.6)	17.4 (2.4)	18.1 (2.7)	17.7 (2.6)
Fiber*, g/d*	31.3 (13)	22.5 (8.9)	26.0 (8.3)	34 (13)	27 (9.9)	30 (8.1)
**Median (IQR)**						
Treats, *serv/wk*	5.2 (5.5)	3.3 (3.3)	4.2 (4.5)	4.2 (4.5)	2.7 (2.7)	3.4 (3.7)
Topping, *serv/wk*	7.9 (15)	4.2 (9.1)	5.6 (12)	7.9 (12.5)	4.5 (8.2)	6.0 (11)
Sweetened beverages, *serv/wk*	1.2 (6.4)	0 (2.1)	0 (4.7)	0 (3.5)	0 (1.4)	0 (2.3)

SMC, Swedish mammography cohort; COSM, Cohort of Swedish men; E%, Percentage of energy intake; IQR, Interquartile range; Serv/wk, Servings per week; Kcal, Kilocalories; SD, Standard deviation.

**Table 2:** Baseline characteristics of the study population according to the added sugar intake categories.

	**Added sugar intake**					
	≤ **5 E%**	>**5–7.5 E%**	>**7.5–10 E%**	>**10–15 E%**	>**15–20 E%**	>**20 E%**
	***N** =* **14,485**	***N** =* **17,779**	***N** =* **14,206**	***N** =* **14,980**	***N** =* **5,365**	***N** =* **2,890**
**N (%)**						
Women	8,351 (57.7)	9,207 (51.8)	6,437 (45.3)	5,990 (40.0)	1,915 (35.7)	1,034 (35.8)
Current smokers	4,110 (28.4)	3,908 (22.0)	2,944 (20.7)	3,353 (22.4)	1,444 (26.9)	851 (29.4)
High alcohol consumption^a^	2,942 (20.3)	3,999 (22.5)	3,005 (21.2)	2,752 (18.4)	843 (15.7)	399 (13.8)
University education	2,869 (19.8)	3,810 (21.4)	2,825 (19.9)	2,434 (16.2)	694 (12.9)	335 (11.6)
High exercise level^a^	1,600 (11.0)	1,945 (10.9)	1,638 (11.5)	1,764 (11.8)	691 (12.9)	374 (12.9)
**Mean (SD)**						
Age, *years*	59.0 (8.73)	59.1 (8.83)	59.9 (9.29)	60.9 (9.58)	61.2 (9.87)	61.1 (9.76)
BMI, *kg/m^2^*	25.4 (3.77)	25.2 (3.47)	25.2 (3.44)	25.3 (3.50)	25.4 (3.54)	25.8 (3.83)
Energy intake, *kcal/day*	2,000 (836)	2,180 (840)	2,290 (868)	2,400 (893)	2,490 (922)	2,540 (1,000)
Added sugar, *E%*	3.6 (1.1)	6.2 (0.7)	8.7 (0.7)	12.1 (1.4)	17.0 (1.4)	25.2 (5.5)
**Median (IQR)**						
Treats, *servings/wk*	2.3 (2.5)	4.0 (3.7)	5.1 (4.7)	5.6 (5.4)	5.5 (6.0)	4.6 (5.7)
Toppings, *servings/wk*	0.7 (2.4)	3.4 (8.0)	8.5 (11.5)	12.4 (17.2)	18.2 (26.7)	17.5 (32.3)
Sweetened beverages, *servings/wk*	0.0 (0.0)	0.0 (1.0)	1.0 (3.5)	4.1 (8.2)	9.8 (11.7)	23.6 (21.4)

E%, Percentage of energy intake; N, Number of participants; Kcal, Kilocalories; SD, Standard deviation; IQR, Interquartile range; ^a^Defined as the highest quintile.

### Added sugar intake and CVD risk

During the follow-up period, 25,739 participants were diagnosed with at least one CVD, including 6,912 cases of ischemic stroke, 1,664 cases of hemorrhagic stroke, 6,635 cases of myocardial infarction, 10,090 cases of heart failure, 1,872 cases of aortic stenosis, 13,167 cases of atrial fibrillation, and 1,575 cases of abdominal aortic aneurysm. The associations between the added sugar intake and the studied outcomes were generally stronger in the first model (adjusted for age, sex, and total energy intake), but attenuated in the second model (additionally adjusted for lifestyle factors), and further attenuated in the main model (additionally adjusted for lifestyle factors, BMI, and dietary factors) (Supplementary Tables S3–S9).

**Figure 2:**
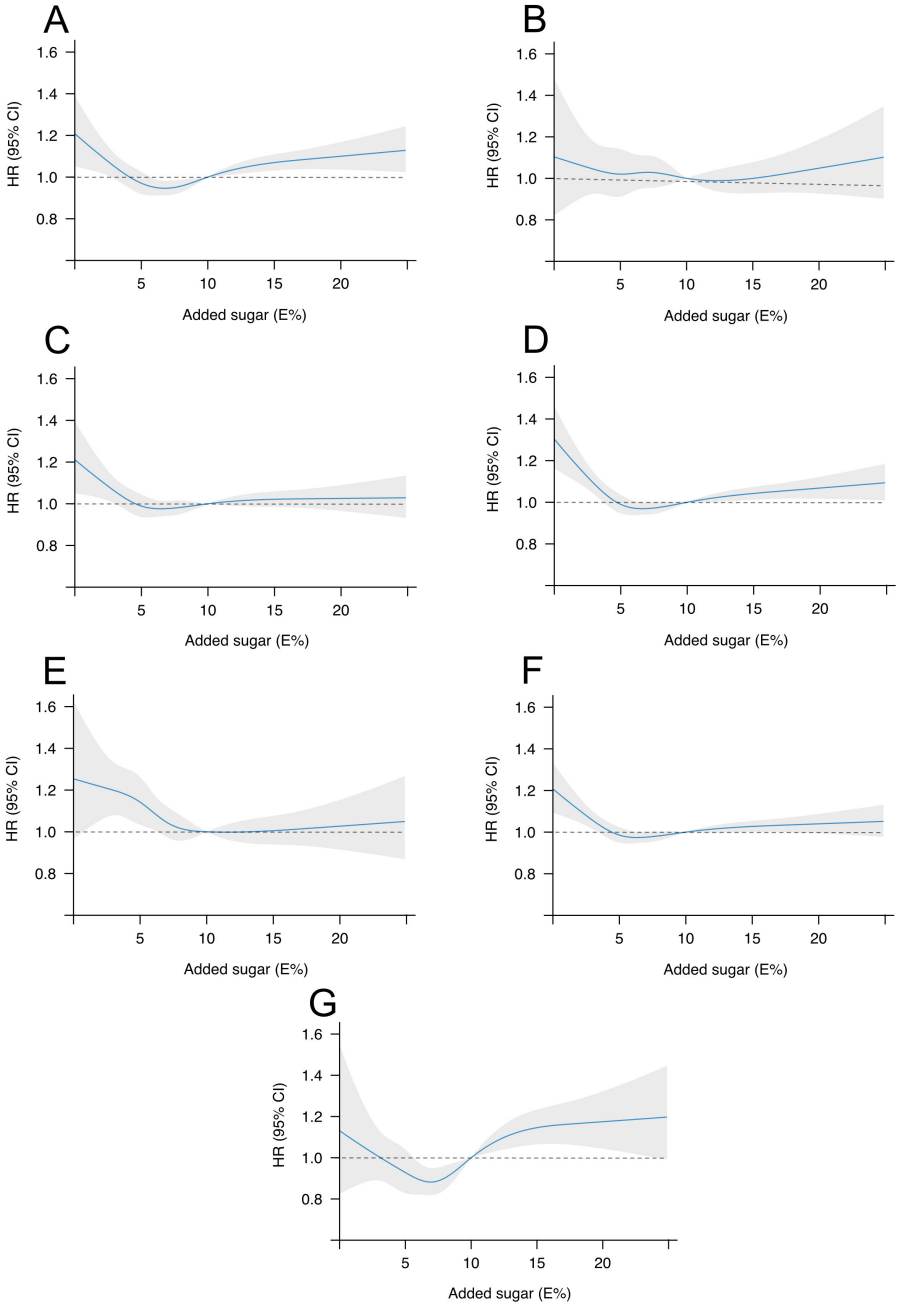
Restricted cubic splines of the associations between added sugar intake as a continuous variable and risk of incident ischemic stroke **(A)**, hemorrhagic stroke **(B)**, myocardial infarction **(C)**, heart failure **(D)**, aortic stenosis **(E)**, atrial fibrillation **(F)**, and abdominal aortic aneurysm **(G)** using a Cox proportional hazards regression with 10 E% as reference value. Adjusted for age, sex, total energy intake, smoking status, educational level, alcohol consumption, walking/bicycling, exercise, BMI, intake of processed meat, coffee, saturated fatty acids, and fiber intake. The solid blue line represents the HR, and the gray area represents the 95 CI. HR, Hazard ratio; CI, Confidence interval; E%, Percentage of energy intake; BMI, Body mass index.

Indications of positive linear associations (*P*_trend_ < 0.01) were found between added sugar intake and risk of ischemic stroke and abdominal aortic aneurysm in the main model. For abdominal aortic aneurysm, a 31% (95% CI: 5–65%) higher risk of abdominal aortic aneurysm was found for added sugar intakes of >20E%, and for ischemic stroke, a 9% (95% CI: 0–19%) higher risk was found for intakes of >15–20 E%, compared to the lowest intake category of ≤ 5 E%. For a majority of the outcomes, however, the highest risks were found in the lowest intake category, whereas the lowest risks were found for low- to moderate intakes. Compared to the lowest intake category ( ≤ 5 E%), added sugar intake of >5–7.5 E% was linked with statistically significant lower risks of ischemic stroke [8% (95% CI: 2-13%)], myocardial infarction [5% (95% CI: 0–11%)], heart failure [9% (95% CI: 5–13%)], aortic stenosis [9% (95% CI: 0–18%)], and atrial fibrillation [7% (95% CI: 3–11%)]. Furthermore, compared to added sugar intakes ≤ 5 E%, lower risks of heart failure and atrial fibrillation were found for intakes of >7.5–10 E% [6% (95% CI: 1–10%), and 4% (95% CI: 0–8%), respectively], as well as of heart failure, atrial fibrillation, and aortic stenosis for intakes of >10–15 E% [5% (95% CI: 0–10%), 4% (95% CI: 0–8%), and 17% (95% CI: 7–26%), respectively]. No associations were found between added sugar intake and hemorrhagic stroke risk (Figure 2, Table 3).

**Table 3:** Associations between added sugar intake and incidence of ischemic stroke, hemorrhagic stroke, myocardial infarction, heart failure, aortic stenosis, atrial fibrillation, and abdominal aortic aneurysm in the study population.

	**Added sugar (E%)**						
	≤ **5 (*****n** =* **14,485)**	>**5–7.5 (*****n** =* **17,779)**	>**7.5–10 (*****n** =* **14,206)**	>**10–15 (*****n** =* **14,980)**	>**15–20 (*****n** =* **5,365)**	>**20 (*****n** =* **2,890)**	**P**_trend_
**Ischemic stroke**							
Cases/PY	1,347/271,034	1,581/337,811	1,407/264,780	1,575/270,859	653/94,558	349/49,295	
Main model	1	0.92 (0.87–0.98)	0.95 (0.90–1.01)	0.98 (0.92–1.04)	1.09 (1.00–1.19)	1.11 (0.99–1.25)	< 0.01
**Hemorrhagic stroke**							
Cases/PY	316/277,117	406/345,228	363/271,256	371/278,347	116/97,658	92/50,819	
Main model	1	1.01 (0.90–1.13)	1.03 (0.91–1.17)	0.97 (0.85–1.11)	0.93 (0.76–1.13)	1.14 (0.89–1.46)	0.93
**Myocardial infarction**							
Cases/PY	1,271/269,512	1,525/335,801	1353/263,271	1,566/268,496	582/93,697	338/48,824	
Main model	1	0.95 (0.89–1.00)	0.95 (0.90–1.01)	0.98 (0.92–1.05)	0.98 (0.90–1.07)	0.99 (0.88–1.11)	0.90
**Heart failure**							
Cases/PY	1,957/270,585	2,319/337,879	2,056/264,967	2,369/270,360	875/94,752	514/48,924	
Main model	1	0.91 (0.87–0.95)	0.94 (0.90–0.99)	0.95 (0.90–1.00)	0.97 (0.90–1.05)	1.03 (0.93–1.13)	0.56
**Aortic stenosis**							
Cases/PY	393/276,564	440/345,058	385/270,996	403/277,867	154/97,354	97/50,677	
Main model	1	0.91 (0.82–1.00)	0.91 (0.82–1.02)	0.83 (0.74–0.93)	0.87 (0.74–1.04)	0.99 (0.79–1.23)	0.15
**Atrial fibrillation**							
Cases/PY	2,577/263,703	3,161/327,847	2,725/256,862	3,026/261,813	1,085/91,790	593/47,972	
Main model	1	0.93 (0.89–0.97)	0.96 (0.91–1.00)	0.96 (0.92–1.00)	0.99 (0.93–1.06)	1.01 (0.93–1.10)	0.66
**Abdominal aortic aneurysm**							
Cases/PY	305/276,866	340/345,173	289/271,533	371/277,755	165/97,294	105/50,587	
Main model	1	0.90 (0.80–1.01)	0.95 (0.84–1.08)	1.09 (0.96–1.24)	1.12 (0.94–1.34)	1.31 (1.05–1.65)	< 0.01

E%, Percentage of energy intake; PY, Person-years; HR, Hazard ratio; CI, Confidence interval; BMI, Body mass index. The associations were investigated using multivariable Cox proportional hazards regressions and the results are expressed as HR with 95% CI, as well as *P*-value for the linear trend using the median values of the added sugar intake categories. All analyses were made according to the main model (adjusted for age, sex, total energy intake, smoking status, educational level, alcohol consumption, walking/bicycling, exercise, BMI, intake of processed meat, coffee, saturated fatty acids, and fiber intake).

### Sugar-sweetened foods and beverages and CVDs

For sweetened beverages, positive linear associations were found for ischemic stroke, heart failure, atrial fibrillation, and abdominal aortic aneurysm (*P*_trend_ < 0.001). Intake of >8 servings per week of sweetened beverages was associated with a 19% (95% CI: 11–27%) higher risk of ischemic stroke, an 18% (95% CI: 11–24%) higher risk of heart failure, an 11% (95% CI: 6–17%) higher risk of atrial fibrillation, and a 31% (95% CI: 15–50%) higher risk of abdominal aortic aneurysm (Supplementary Tables S3–S9).

For treats, negative linear associations were found with all outcomes (*P*_trend_ < 0.01), with the highest risks being found among consumers of ≤ 2 servings/week (Supplementary Tables S3–S9).

For toppings, negative linear associations were found with heart failure, and aortic stenosis, while a positive linear association was found with abdominal aortic aneurysm (*P*_trend_ < 0.01). Specifically, ~10% lower risks of heart failure were found for all intake categories compared to the lowest intake category ( ≤ 2 servings/week). A 34% (95% CI: 18–51%) higher risk of abdominal aortic aneurysm was found for the highest intake category of toppings (>28 servings/week) compared to the lowest intake category. For aortic stenosis, 16% (95% CI: 5–25%), 20% (95% CI: 9–29%), and 15% (95% CI: 3–25%) lower risks were found for toppings intakes of >7–14, >14–28, and >28 servings/week, respectively, compared to the lowest intake category (Supplementary Tables S3–S9).

### Sensitivity analyses

**Figure 3:**
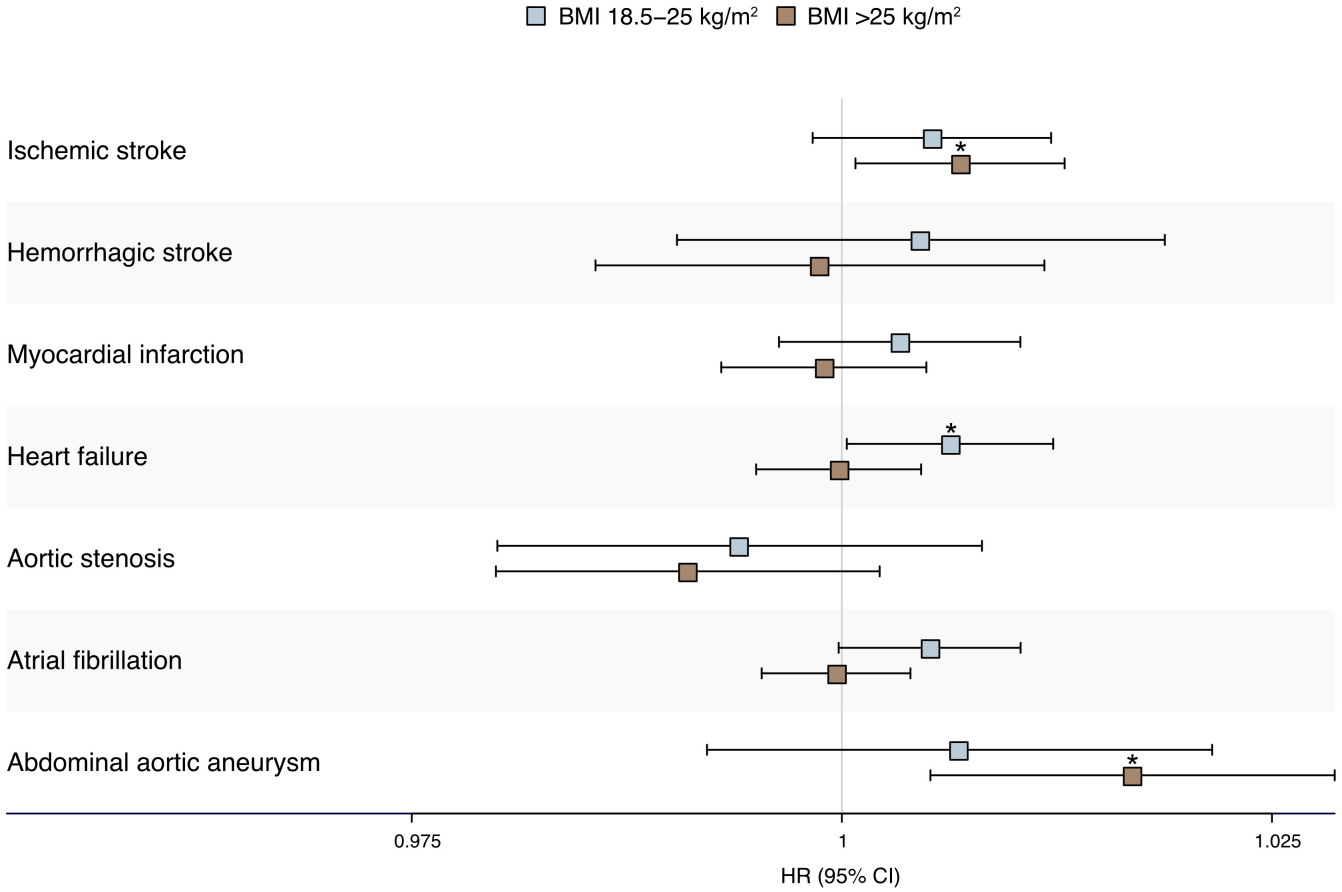
Forest plot describing the HR (95% CI) per E% increase of added sugar intake for incident cardiovascular disease in participants stratified by BMI 18.5–25 kg/m^2^ and 25 kg/m^2^. *Statistically significant association. CI, Confidence interval; HR, Hazard ratio.

No interactions were found between sex and added sugar intake for the studied outcomes (*P* > 0.05). Interactions were found between added sugar intake and BMI for atrial fibrillation and heart failure (*P* < 0.05). Higher added sugar intake was associated with higher risks of abdominal aortic aneurysm and ischemic stroke in individuals with BMI >25 kg/m^2^, while higher added sugar intake was associated with a higher risk of heart failure in individuals with BMI 18.5–25 kg/m^2^ (Supplementary Tables S10, S11, Figure 3).

The associations for sugar-sweetened beverage and artificially sweetened beverage intake were studied separately among the 42,327 participants who had answered the 2009 dietary assessment. Among these participants, 10,843 individuals reported consumption of at least 1 serving/week of sugar-sweetened beverages, and 5,727 participants reported consumption of at least 1 serving/week of artificially sweetened beverages. Positive linear associations were observed between intake of artificially sweetened beverages and risk of ischemic stroke (*P*_trend_ < 0.01) and heart failure (*P*_trend_ < 0.01) (Supplementary Table S12), while intake of sugar-sweetened beverages was not associated with CVD risk (Supplementary Table S13).

To take comorbidity into consideration, all participants with prior incidence of the other studied CVDs during the follow-up period were excluded for each studied outcome. This resulted in slightly attenuated associations between added sugar intake and most of the studied outcomes; however, the tendencies and directions of the previously linear associations remained, as positive linear associations were observed for ischemic stroke (*P*_trend_ = 0.05) and abdominal aortic aneurysm (*P*_trend_ < 0.01), while a negative linear association was observed between added sugar intake and incident aortic stenosis (*P*_trend_ = 0.04) (Supplementary Table S14).

The risk estimates did not differ notably when testing the associations for added sugar and sugar-sweetened food and beverage intake with energy adjustments using the nutrient density method or the residual method for added sugar intake, nor between the standard multivariate method, nutrient density method, or residual method for sugar-sweetened foods and beverages (Supplementary Tables S15–S18).

When studying the associations between added sugar and CVD incidence using the 1997 dietary data only, the results were similar to the main results but with lower precision (Supplementary Table S19). Similarly, excluding cases of the studied outcomes within the first 3 years of the follow-up period did not alter the results (Supplementary Table S20).

When only including the participants with complete data for sugar-sweetened foods and beverages and categorical covariates, the overall results remained similar to the main analyses (Supplementary Tables S21–S24).

## Discussion

The findings of this study on the associations between added sugar and CVD risk indicate that the associations vary depending on the disease and the source of added sugar, demonstrating the importance of studying them separately. There were statistically significant linear associations between total added sugar intake and ischemic stroke and abdominal aortic aneurysm, but the highest risk of most of the studied outcomes were found in the lowest intake category. High intake of sweetened beverages was associated with higher risk for most of the studied outcomes, for which positive linear associations were found. In contrast, a low intake of treats was associated with a higher risk of all the studied outcomes. Incorporating the time-updated 2009 information in the analyses resulted in higher precision of the results.

Very few studies have investigated the associations between added sugar intake and incidence of specific CVDs, and this study is, to the best of our knowledge, the first one to investigate the associations with many of the studied outcomes such as for example abdominal aortic aneurysm and atrial fibrillation. Results from the Women's Health Initiative did not show associations between added sugar intake and incident heart failure nor stroke (14). In the Swedish Malmö Diet and Cancer Study, the associations for sugar intake also varied depending on CVD disease and source of added sugar (13, 21). In that study, U-shaped associations were found between added sugar intake and overall stroke risk (13). In a study of UK biobank participants, free sugar intake was linearly associated with overall stroke risk (15). In the current cohort we divided stroke into subtypes and found a positive linear association with ischemic stroke (*P*_trend_ < 0.01) but no associations with hemorrhagic stroke. The non-linear associations between added sugar intake and atrial fibrillation and aortic stenosis were comparable between the Malmö Diet and Cancer Study and this study, with the highest relative risk found in the lowest intake category for both outcomes.

Although mechanisms explaining the associations between added sugar intake and CVD risk are not well-established, several biological mechanisms have been proposed. One hypothesis is based on the distinct metabolism of fructose, a component of the common added sugar sucrose. Fructose is metabolized in the liver and converted to glycerol-3-phosphate, which serves as a backbone for triacylglycerol synthesis, leading to increased triacylglycerol synthesis and ultimately increased CVD risk (20, 30). It is important to highlight that the notion of adverse metabolic effects of fructose has been questioned, as many studies on the topic refer to very high intakes of fructose (>95th percentile) which do not reflect the metabolic effects of normal dietary intakes (31, 32). The EFSA scientific opinion on a tolerable upper intake level for dietary sugars does however state that evidence from prospective cohort studies suggest positive and causal relationship between the intake of fructose and risk of cardiovascular diseases, albeit with a low level of certainty (6).

Relationships between added sugar intake and CVD risk factors such as dyslipidaemia (5, 6), hypertension (6), and obesity, have been shown in both randomized controlled trials and observational studies, and could help explain the associations found between added sugar intake and some of the studied CVDs. Although the mentioned CVD risk factors affect all of the studied CVDs to some extent, one explanation for the findings for the different CVDs may be that the risk factors affect the diseases to varying degrees. For example, aortic stenosis can be caused by congenital heart defects and hemorrhagic stroke can be caused by head injuries, whereas ischemic stroke and abdominal aortic aneurysm might be more dependent on atherosclerotic processes. This could result in the potential effect of dietary factors on disease incidence being smaller and more difficult to discern for some of the studied outcomes. This would however not explain the lack of associations found with for example myocardial infarction, as it is also a highly atherosclerotic disease. Consequently, more research is needed to gain deeper understanding of the possible associations and mechanisms of added sugar intake and CVD risk.

The results for the associations between added sugar and some CVDs in individuals with overweight and obesity in this study suggest that BMI may be an effect modifier of the associations, which could explain some of the discrepancies between studies as the rates of overweight or obesity vary in different populations. The role of BMI as a potential effect modifier of the associations between added sugar intake and CVD risk consequently warrants further investigation. Further, although consumption of < 5 E% of added sugar was associated with a higher risk of several CVDs, it is important to highlight that there are no well-established biological mechanistic explanations for these associations. Findings from the Australian Health Survey 2011–2012 (weighted *n* = 6,150) did however indicate that added sugar intakes below 5 E% were associated with lower micronutrient intakes (33), and it is also possible that the added sugar is substituted by other nutrients such as for example saturated fats. Both of these factors could contribute to adverse cardiovascular health. On the other hand, results from two Swedish populations (*n* = 12,238 and *n* = 1,797) indicated an inverse association between added sugar intake and micronutrient intake, contradicting the micronutrient dilution theory (34). Finally, methodological limitations such as residual confounding and misreporting could affect the results and explain some of the findings. The overall findings based on CVD health do not support lowering the recommendations for added sugar intake to below 5 E%, although they do indicate lower risks of CVDs among consumers of >5–7.5 E% than among consumers of 10 E% of added sugar.

The clear associations between overall sweetened beverage intake and a higher risk of several CVDs (i.e., ischemic stroke, heart failure, atrial fibrillation, and abdominal aortic aneurysm) found in this study are supported by previous findings. However, the findings specifically for artificially sweetened beverages and sugar-sweetened beverages are not as congruous with existing literature which points toward them being associated with CVD risk as well as risk markers for CVD (6, 35–37). In this study, however, higher intake of artificially sweetened beverages was associated with increased risks of ischemic stroke and heart failure, while no associations were found between intake of sugar-sweetened beverages and CVD risk. This could be explained by a relatively low frequency of sugar- and artificially sweetened beverage consumption, as 10,843 (26%) and 5,727 (14%) individuals, respectively, reported consumption of at least 1 serving/week. Only 2,499 (6%) individuals consumed >8 servings/week of sugar-sweetened beverages and 1,660 (4%) individuals consumed >8 servings/week of artificially sweetened beverages. To put it into perspective, a review article reported an 8% increased CVD risk with each additional consumed serving of sugar-sweetened beverages per day (38), thus it is possible that there were too few sugar- and artificially sweetened beverage consumers, and in particular, too few high-frequency consumers, to detect similar associations. It should also be mentioned, however, that it has been suggested that associations found with artificially sweetened beverages could be affected by reverse causality (36). It should also be noted that while some literature suggests that consumption of artificial sweeteners could impact cardiovascular health negatively by alteration of the gut microbiota, the collected body of evidence does not indicate adverse effects of artificially sweetened beverage intake on CVD risk (39).

The findings for treats are consistent with previous findings in the Malmö Diet and Cancer Study, in which the highest risks of stroke, coronary events, atrial fibrillation, and aortic stenosis were found in the lowest intake category ( ≤ 2 servings/week) (13). Similar findings were reported for all-cause mortality in the Malmö Diet and Cancer Study and the Northern Swedish Health and Disease Study, where inverse associations were reported with intake of treats (21). The findings for toppings are less uniform, as the results from this study indicated positive associations between intake of toppings and risk of heart failure and aortic stenosis, and negatively associated with risk of abdominal aortic aneurysm, while no associations were found with intake of toppings and CVD risk in Malmö Diet and Cancer Study (13). Given that this study has more than twice as many participants as the Malmö Diet and Cancer Study, it is possible that the discrepancy could be due to a higher power to detect associations.

The findings of this study indicate that not all sources of added sugar are equally harmful to health, as sugar-sweetened beverages are the primary source of added sugar associated with increased CVD risk. Possible explanations of the discrepancies between the associations for sweetened beverages and treats and toppings include liquid calories providing lower satiety and insufficient compensatory reduction of caloric intake, thus promoting overweight and obesity, which are established risk factors for CVD (40). It should be noted that there are currently no mechanistic explanations for the negative linear associations found for treats. One aspect to take into consideration is however that there is a social tradition of “fika” in Sweden, where people get together with friends, relatives, or coworkers for coffee and pastries (41). Thus, one could hypothesize that the intake of treats is part of many people's everyday lives without necessarily being related with overall poor dietary or lifestyle patterns, and that it might be a marker of social life. Consumption of sweetened beverages, one the other hand, has been linked to lower overall dietary quality (42).

Despite adjusting for plausible confounders, we cannot rule out the presence of confounders due to the observational nature of this study and the complexity of diet as an exposure. The confounders are also likely to differ between the outcomes. In this study, while adjustments were made for processed meat consumption, a significant contributor to sodium intake in Sweden, direct sodium intake was not accounted for. The FFQ used in the cohorts did not account for salt used in cooking or at the table, and therefore, dietary sodium intake could not be estimated. Moreover, existing literature indicates poor agreement between sodium intake estimated from FFQs and urinary sodium excretion (43), highlighting the need for future research to incorporate more accurate measures, such as urinary sodium excretion, to more precisely assess the impact of sodium intake as a potential confounding factor. Further, we did not adjust for factors accounting for social networks, which is something that should be further explored in future studies. As inverse associations have been found between intake of treats and CVD incidence in these cohorts as well as others (21), intake of treats should be studied closer with regards to biological mechanisms as well as possible correlations with other factors that could impact the observed associations. Other limitations of this study include the self-reported dietary information, which may introduce some misreporting and, as a result, misclassification of exposure. Although we excluded individuals deemed to have implausible energy intakes, some misclassification of exposure may remain. Additionally, approximately 39% of the participants did not complete the 2009 assessment, meaning that information on diet and covariates was only available during baseline for those participants.

In comparison to the national mean added sugar intake of 9.6 E% in Sweden in 2010–2011 (8), the mean added sugar intakes in the study population were lower, estimated to be 9.0 E% in 1997 and 6.7 E% in 2009. In this study population, the intakes of sugar-sweetened foods and beverages were also lower in 2009 than in 1997, despite the national intake levels of sweetened beverages and treats increasing throughout the same period (44). Aside from an actual difference in sugar intake between 1997 and 2009 in SMC and COSM, one possible explanation for this difference is that the general perception of the health effects of added sugar differed during those time periods, and thus it could be theorized that misreporting of added sugar was more prevalent in 2009 than in 1997. Despite this, the precision of the results was higher when incorporating the updated information from 2009.

Strengths of this study include the large study population of 69,705 individuals as well as the repeated assessments for the majority of the participants, which allowed us to gain insights into the participants' diets at two separate time-points, leading to higher precision and likely reducing misclassification of exposure, though this cannot be completely ruled out. Finally, because the associations between added sugar intake and different CVD outcomes can vary, this study investigated the risk of the CVDs separately. We also studied added sugar intake as a whole and in groups of sugar-sweetened foods and beverages, providing higher granularity and further insights into how different forms of added sugar intake associate with CVD risk. Due to the large number of statistical tests performed in this study, there is a risk of chance findings. The findings of this study could help inform future dietary guidelines and recommendations which could guide policies to reduce CVD incidence and ultimately improve public health.

The results of this study are consistent with previous findings in another Swedish middle-aged population (13), but the associations between added sugar intake and CVD risk cannot necessarily be generalized to other populations as the consumption patterns of added sugar and sugar-sweetened foods and beverages may vary across countries and age groups. More research is needed to discern whether the observed associations, and the findings for treats in particular, can be replicated in other populations, and future research should be conducted to explore potential mechanisms and associations with intermediary risk factors. To acquire a better understanding of the associations between added sugar intake and CVD risk, other methods such as using objective biomarkers or Mendelian randomizations could be used to investigate any causal links between added sugar intake and CVD risk in observational studies.

## Conclusion

According to the findings of this study, the associations between added sugar intake and CVDs vary substantially depending on the disease and source of added sugar. Finally, the findings of this study emphasize the adverse health effects associated with sweetened beverage consumption and indicate higher CVD risks among individuals with low intakes of treats.

## Data Availability

The data is accessed through SIMPLER (Swedish Infrastructure for Medical Population-based Life-course and Environmental Research) and since it is an individual-based database and biobank, ethical approval is needed to get access to the data. Consequently, for ethical reasons, the data can't be openly accessed and any requests to access these datasets should be directed to https://www.simpler4health.se/w/sh/en/researchers/data-access.
